# Supplementary material for: Exploring the Design Space of Machine Learning Models for Quantum Chemistry with a Fully Differentiable Framework
Source: J Chem Theory Comput. 2025 Jun 25;21(13):6505–16. doi: 10.1021/acs.jctc.5c00522 (PMC12243086; doi:10.1021/acs.jctc.5c00522)
Supplement: Supplementary file 1 [file ct5c00522_si_001.pdf]

Supporting Information: Exploring the design space of machine-learning models for quantum chemistry with a fully differentiable framework

Divya Suman,[†,¶] Jigyasa Nigam,[*,†,¶] Sandra Saade,[†] Paolo Pegolo,[†] Hanna Türk,[†] Xing Zhang,[‡] Garnet Kin-Lic Chan,[‡] and Michele Ceriotti[*,†]

†*Laboratory of Computational Science and Modeling, Institut des Matériaux, École Polytechnique Fédérale de Lausanne, 1015 Lausanne, Switzerland*

‡*Division of Chemistry and Chemical Engineering, California Institute of Technology, Pasadena, CA 91125, USA*

¶*These authors contributed equally to this work*

E-mail: jnigam@mit.edu; michele.ceriotti@epfl.ch

1 Dataset generation

To train our models, we use a subset of molecules from the QM7b dataset[S1] that contains only H, C, N, and O atoms. Specifically, we select 1000 molecules from the QM7b dataset according to Farthest Point Sampling (FPS)[S2,S3] as implemented in `scikit-matter`,[S4] which incrementally selects structures from a dataset to maximize a specified distance metric on a manifold. In this case, we maximize the distance between SOAP power spectrum features[S5] that characterize the local atomic environments in each molecule. Fig. S1 visualizes the

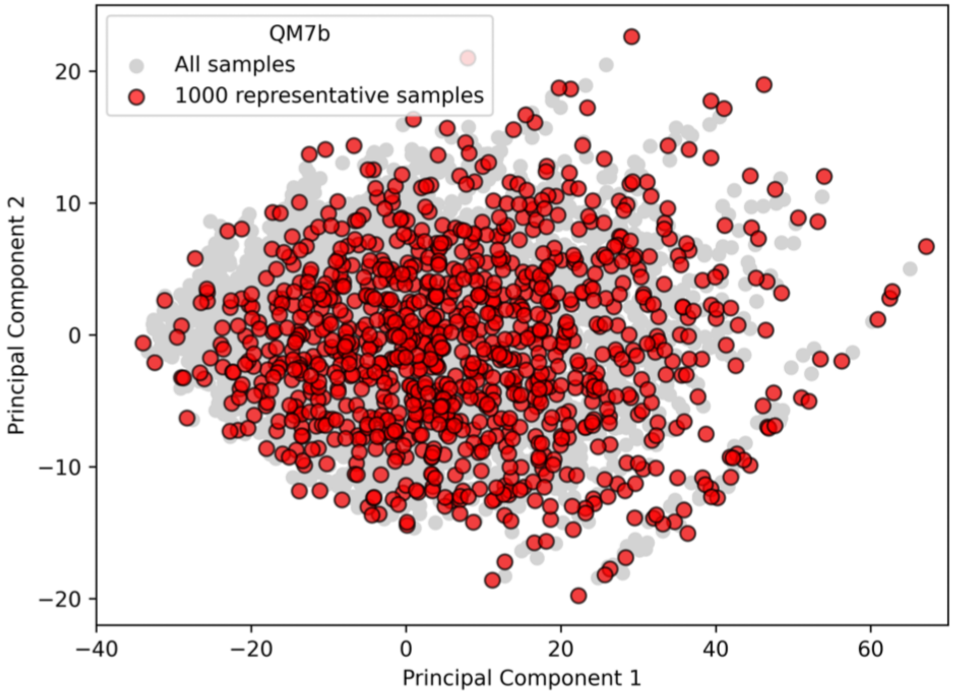

Figure S1: Two-dimensional representation of the QM7b and the CHNO subset of the QM9 datasets (gray). The red dots represent the structures selected by the FPS procedure.

selected structures (in red) projected onto the first two principal components of the features.

To evaluate the model performance, we randomly select 200 molecules from a 20,000-molecule subset[S6] of the QM9 dataset,[S7] composed of the same chemical elements as in the training set. The test set thus reflects the natural distribution of molecular structures within QM9, and is indicative of an unbiased estimate of the model transferability to unseen datasets. We additionally use the showcase dataset[S8,S9] comprising polyalkene, polyacene chains, and polyenoic acid series to assess the extrapolative power of our model in predicting the dipole moments and polarizabilities of more complex, unseen molecules.

1.1 Electronic structure calculations

For the QM7, QM9, showcase `AlphaML` and `MuML` datasets, we performed Kohn–Sham density functional theory (KS-DFT) calculations with PySCF,[S10] using the B3LYP functional.[S11] For each molecular geometry, we computed the Fock and overlap matrices along with other

electronic properties such as density matrices, dipole moments, and polarizabilities, using both STO-3G and def2-TZVP basis sets. The STO-3G minimal basis contains 1s orbitals for H, and 1s, 2s, and 2p orbitals for C, N, and O atoms, while the def2-TZVP basis set consists of 1s, 2s, 2p, 3s, 3p orbitals for H and 1s, 2s, 2p, 3s, 3p, 3d, 4s, 4p, 4d, 4f and 5s orbitals for C, N, and O.

For the condensed phase Hamiltonian dataset, we randomly selected 23 structures from a graphene dataset reported in Ref. S12. For these selected structures, we performed DFT calculations using CP2K,[S13] with the Perdew-Burke-Ernzerhof (PBE) exchange-correlation functional[S14] using both the SZV and DZVP basis sets with GTH pseudopotentials.[S15] The SZV minimal basis set contains only one basis function per valence orbital, while the DZVP basis set includes two functions per valence orbital as well as additional polarization functions. All calculations were performed at the Gamma point in the Brillouin zone on a 72-atom supercell.

2 Feature hyperparameters and training details

In this section, we describe the training details of the models presented in the main text. We use the `featomic`[S16] library to compute both atom-centered and pair features. We use a specific kind of atom-centered density correlation descriptor,[S5,S17,S18] corresponding to two-neighbor (or three-body) correlations, denoted by $\boldsymbol{\xi}^{\sigma\lambda\mu}_{A_i}$. These features rely on tensor products of atom densities $c_{nlm}(A_i)$, which is defined as

$$c_{anlm}(A_i) = \sum_{j\in A_i} c_{anlm}(A_{ij})\delta_{aa_j}. \tag{1}$$

$c_{anlm}(A_{ij})$ denote the pair-density coefficients, describing the position of atom j relative to atom i on a discretized basis of radial functions R_{nl} and angular functions (spherical harmonics) $Y_l^m(\hat{r})$,

$$c_{anlm}(A_{ij}) = R_{nl}(r_{ji})Y_l^m(\hat{\mathbf{r}}_{ji}). \tag{2}$$

The sum in Eq. (1) is usually restricted to neighboring atoms j within a local environment centered on atom i (A_i), usually limited within a spherical cutoff r_{cut}. Note that we only include neighboring atoms which are of the same chemical species as the chemical basis a. $\boldsymbol{\xi}^{\sigma\lambda\mu}_{A_i}$, is then obtained as the symmetrized tensor product,

$$\boldsymbol{\xi}^{\sigma\lambda\mu}_{A_i} = \sum_{mm'} \langle lm; l'm' | \lambda\mu \rangle \; c_{anlm}(A_i) \, c_{a'n'l'm'}(A_i) \tag{3}$$

where boldface indicates the dimensions of the feature, enumerated by a, a', n, n', l, l', that are contracted with the model weights. σ tracks the parity of the equivariant features under inversion and is equal to $(-1)^{l+l'+\lambda}$. On the other hand, pair (two-center) features, which simultaneously describe the atom pair (ij), are obtained through tensor products of the pair density (2) with the atom-centered descriptor on atom i,

$$\boldsymbol{\xi}^{\sigma\lambda\mu}_{A_{ij}} = \sum_{mm'} \langle lm; l'm' | \lambda\mu \rangle \; c_{anlm}(A_i) \, c_{a'n'l'm'}(A_{ij}), \tag{4}$$

describing the correlations of the pair of atoms i, j with the neighbors of i. More details about the construction and symmetrization of pair features may be found in Ref. S19.

Although the cutoff, r_{cut}, is a hyperparameter, it is usually tuned to emphasize the locality of atomic environments, which implies that atoms i and j separated by distances larger than the cutoff will not have any associated features, and hence all their Hamiltonian blocks will be predicted to be identically zero. This can be addressed by computing $c_{nlm}(A_{ij})$ in (1) and (2) with different cutoffs and combining them, as in (4).

Each atomic position was represented with a Gaussian smearing of width 0.3 Å. To compute the density expansion coefficients in (1), we used 6 radial basis functions and spherical harmonics up to $l = 4$. We used a cutoff of 3 Å to compute the atom-centered features, and a cutoff of 5 Å for the two-center features. For smoothness, we also used a shifted cosine cutoff function that smoothly decays over 0.1 Å.

The features so computed are used as inputs to a symmetry-adapted ridge regression

model with cross-validation (RidgeCV) to predict the elements of the Hamiltonian matrix in the STO-3G basis. We use regularization (α) values between 10^{-8} to 10^{3} and a three-fold cross-validation (cv=3) to avoid any over-fitting. This (direct) model is further fine-tuned to optimize the loss on desired properties by backpropagating the loss on them (indirect learning). Here we describe the loss function for one of the models used in the main text, $(\varepsilon, \boldsymbol{\mu}, \boldsymbol{\alpha}, \boldsymbol{B})$,

$$\begin{aligned}\mathcal{L}_{\varepsilon,\boldsymbol{\mu},\boldsymbol{\alpha},\boldsymbol{B}} = & \frac{\omega_\varepsilon}{N}\sum_{n=1}^{N}\frac{1}{O_n}\sum_{o=1}^{O_n}\left(\varepsilon_{no}-\tilde{\varepsilon}_{no}\right)^2 + \frac{\omega_\mu}{N}\sum_{n=1}^{N}\frac{1}{N_{A_n}^2}\sum_{m=1}^{N_A}\left(\mu_{nm}-\tilde{\mu}_{nm}\right)^2 \\ & + \frac{\omega_\alpha}{N}\sum_{n=1}^{N}\frac{1}{N_{A_n}^2}\sum_{m=1}^{N_A}\left(\alpha_{nm}-\tilde{\alpha}_{nm}\right)^2 + \frac{\omega_B}{N}\sum_{n=1}^{N}\frac{1}{N_B}\sum_{m=1}^{N_B}\left(B_{nm}-\tilde{B}_{nm}\right)^2\end{aligned} \tag{5}$$

where N is the number of training points, O_n is the number of MO orbitals in the nth molecule, N_{A_n} is the number of atoms in the nth molecule, N_B is the number of elements in the Mayer bond order matrix. ε_{no}, μ_{nm}, α_{nm} and B_{nm} are the target MO energy, dipole moment, polarizability and Mayer bond order, respectively, while the tilde decorations over the same represent the corresponding predicted values. The hyperparameters ω_ε, ω_μ, ω_α and ω_B control the influence of different losses during training, and adjusted to ensure that all these MSE losses are comparable in scale.

We use $\omega_\varepsilon = 10^4$, $\omega_\mu = 10^3$, $\omega_\alpha = 10^2$ and $\omega_B = 10$ for training. The data described in 1 is divided into training (70%), validation (20%), and test (10%) subsets. We use a batch size of 100 to optimize computational efficiency and model convergence. The model is trained in `PyTorch`[S20] using the Adam optimizer with a learning rate (LR) of 1e-3. The LR is controlled by a scheduler, that reduces the LR by a factor of 0.8 if the validation loss does not improve for 20 consecutive epochs. Each training epoch takes approximately 4 to 4.5 minutes, and we train the model for up to 1000 epochs, stopping when the loss vs. epoch curve indicates saturation. This training strategy ensures a balance between model performance and computational efficiency. To ensure robust error estimation, we train our

models using three different train-test splits and report the mean and standard deviation of the model performance across these different models.

3 Comparison of random vs STO-3G initialization in indirect learning

To evaluate the significance of the initialization point in the training of indirect effective Hamiltonian models, we compare the performance of a model that is initialized with random weights against a model where the parameters are initialized to the weights obtained from a direct **H** model trained to reproduce the STO-3G Hamiltonian matrix elements (i.e. weights from Eq. 7 of the main text). The results for the QM7 test dataset are summarized in Table S1. We observe that the indirect learning task is particularly sensitive to initialization

Table S1: Comparison of mean absolute errors (MAE) between a model using a random initialization and a model initialized using the ridge-fitted weights of an STO-3G Hamiltonian matrix (STO-3G init.) for the QM7 test dataset.

**Quantities**	**Random init.** $(\varepsilon, \boldsymbol{\mu}, \boldsymbol{\alpha}, \mathbf{B})$	**STO-3G init.** $(\epsilon, \boldsymbol{\mu}, \boldsymbol{\alpha}, \mathbf{B})$
$\mathrm{MAE}_{\varepsilon}$ (meV)	54637.14	314.29
$\mathrm{MAE}_{\mathrm{gap}}$ (meV)	6629.37	584.25
MAE_{μ} (mD/atom)	159.01	1.76
MAE_{α} (Å^3/atom)	32.45	0.12
$\mathrm{MAE}_{\mathbf{B}}$ (-)	16.14	3.59

on account of to two factors. First, the non-convexity of the learning problem induced by nonlinear operations involved in diagonalization and property evaluation, even when the Hamiltonian is linearly parameterized, and second, the severe under-determination of the indirect learning problem, which leads to overfitting and convergence to nonphysical local minima. Initialization with a physically meaningful Hamiltonian bypasses these issues and dramatically improves model performance across all predicted quantities.

4 Effect of basis set parametrization on model performance

To further examine the role of initialization and parametrization in indirect Hamiltonian models, we report an additional experiment in which we optimize an effective Hamiltonian that is bigger than the minimal STO-3G but still much smaller than the def2-TZVP basis. Specifically, we use a 6-31G basis (which contains 1s and 2s orbitals for H, and 1s, 2s, 2p, 3s and 3p orbitals for C, N, and O atoms), rather than the minimal STO-3G basis used throughout the main body of this work. Following exactly the same procedure, we first train a direct **H** model (ridge regression) to match the 6-31G Hamiltonian matrix elements, which is then fine tuned to reproduce on all quantitites $(\varepsilon, \boldsymbol{\mu}, \boldsymbol{\alpha}, \boldsymbol{B})$ computed in the def2-TZVP basis. To ensure a fair comparison of this model with the one initialized with a ridge regression on STO-3G Hamiltonian, we used the same training parameters and the same subset of the QM7 dataset for both models.

Table S2: Comparison of mean absolute errors (MAE) between the models using STO-3G (STO-3G init.) and 6-31G basis (6-31G init.) as the effective basis (EB) for the QM7 test dataset.

**Test dataset**		**STO-3G init.** $(\varepsilon, \boldsymbol{\mu}, \boldsymbol{\alpha}, \mathbf{B})$	**6-31G init.** $(\varepsilon, \boldsymbol{\mu}, \boldsymbol{\alpha}, \mathbf{B})$
QM7	$\mathrm{MAE}_{\varepsilon}$ (meV)	314.29	262.54
	$\mathrm{MAE}_{\mathrm{gap}}$ (meV)	584.25	341.94
	MAE_{μ} (mD/atom)	1.76	1.51
	MAE_{α} (Å^3/atom)	0.12	0.04
	$\mathrm{MAE}_{\mathbf{B}}$ (-)	3.59	1.35

Table S2 summarizes the MAEs on target properties for QM7 test structures, using indirect models with both STO-3G and 6-31G as the effective basis. The model that refines an intermediate Hamiltonian on the 6-31G basis outperforms its STO-3G counterpart across most properties. The most notable improvement is observed in the prediction of polarizabil-

ity. This improvement highlights the benefit of using a more expressive basis set for model parametrization, especially when capturing properties sensitive to the completeness of the basis.

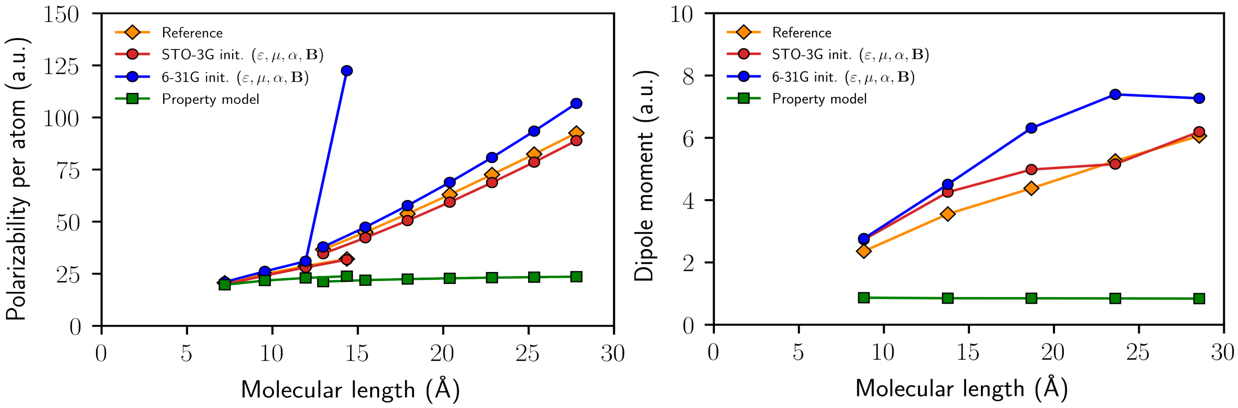

Figure S2: Predicted polarizability per atom for the polyalkene and polyacene series (left) and dipole moment of the polyenoic acid series (right) as a function of molecular length. The yellow line denotes the reference quantum mechanical values computed using the def2-TZVP basis set. The red line corresponds to the model that indirectly optimizes an effective minimal STO-3G basis Hamiltonian, while the blue line shows the model with 6-31G as the effective basis. For comparison, green lines represent property-specific models trained directly on the observables (e.g., AlphaML, MuML analogues). While the effective 6-31G model performs better on small molecules, it exhibits substantial overestimation in the extrapolative regime. In contrast, the STO-3G-based model generalizes more robustly, approximating the reference values more closely across molecular sizes.

However, as shown in Fig. S2, this advantage does not carry over to extrapolative regimes. When tested on out-of-distribution structures, i.e. polyalkenes and polyacenes series as well as the dipole moments for the polyenoic series, the effective 6-31G model exhibits poorer extrapolation. It tends to overestimate both dipole moments and polarizabilities with increasing molecular size, diverging more substantially from the reference calculations than the corresponding effective STO-3G model. This tradeoff can be attributed to the increased representational capacity of the 6-31G model, which allows it to fit more closely to the training data but also increases its susceptibility to overfitting, especially when the training set is limited in size and diversity. This suggests that while richer intermediate basis sets (such as 6-31G) may benefit in-domain accuracy, they require larger and more diverse

training datasets to fully realize their potential in extrapolative regimes. Future work will explore whether regularization strategies can mitigate this tradeoff and better leverage the expressivity of larger basis sets.

5 Supplementary Tables

Table S3: Mean absolute errors (MAEs) and standard deviations for various *simple* indirect models, that employ the STO-3G basis for both model training and target calculations, across different datasets (QM7, QM9, Polyenoic acid series and Polyalkenes/acenes series). The evaluated properties include the MO energies (ε), MO energies upto the occupied orbital (ε_{occ}), HOMO-LUMO gap (Δ), dipole moment ($\boldsymbol{\mu}$), polarizability ($\boldsymbol{\alpha}$) and Mayer bond order ($\mathbf{B}$). The "property model" row represents the performance of property-specific models, while subsequent rows correspond to multi-task effective Hamiltonian models trained on increasing numbers of properties.

dataset	Model	ε (meV)	ε_{occ} (meV)	Δ (meV)	μ (mD/atom)	α (Å^3/atom)	$\mathbf{B}$ (-)
QM7	property model	-	-	-	1.10 (±0.083)	0.05 (±0.002)	-
	(ε)	139.06 (±6.886)	148.87 (±10.011)	224.15 (±8.826)	6.45 (±0.361)	0.04 (±0.001)	1.67 (±0.168)
	($\varepsilon, \boldsymbol{\mu}$)	153.80 (±7.084)	159.76 (±10.297)	237.15 (±24.433)	0.97 (±0.105)	0.02 (±0.001)	0.80 (±0.105)
	($\varepsilon, \boldsymbol{\mu}, \boldsymbol{\alpha}$)	308.69 (±11.376)	320.04 (±21.719)	452.94 (±40.481)	1.40 (±0.124)	0.01 (±0.001)	0.57 (±0.086)
	($\varepsilon, \boldsymbol{\mu}, \boldsymbol{\alpha}, \mathbf{B}$)	285.64 (±21.378)	282.02 (±36.444)	425.80 (±50.928)	1.27 (±0.111)	0.01 (±0.003)	0.20 (±0.021)
QM9	property model	-	-	-	2.32 (±0.025)	0.08 (±0.002)	-
	(ε)	232.69 (±3.156)	252.84 (±4.435)	384.53 (±13.905)	9.65 (±0.342)	0.05 (±0.001)	2.12 (±0.174)
	($\varepsilon, \boldsymbol{\mu}$)	247.82 (±1.290)	264.93 (±2.661)	399.18 (±10.153)	2.25 (±0.046)	0.04 (±0.001)	0.92 (±0.086)
	($\varepsilon, \boldsymbol{\mu}, \boldsymbol{\alpha}$)	379.44 (±10.818)	375.51 (±12.302)	440.98 (±36.847)	2.68 (±0.154)	0.02 (±0.001)	0.74 (±0.089)
	($\varepsilon, \boldsymbol{\mu}, \boldsymbol{\alpha}, \mathbf{B}$)	356.93 (±6.735)	361.02 (±10.480)	518.19 (±58.311)	2.55 (±0.093)	0.03 (±0.001)	0.39 (±0.019)
Polyenoics	property model	-	-	-	6.91 (±0.040)	1.21 (±0.009)	-
	(ε)	244.35 (±4.208)	270.35 (±3.282)	760.99 (±42.843)	13.20 (±0.804)	0.48 (±0.051)	3.87 (±0.221)
	($\varepsilon, \boldsymbol{\mu}$)	236.78 (±9.954)	258.15 (±17.781)	714.58 (±55.435)	2.00 (±0.191)	0.25 (±0.050)	1.33 (±0.112)
	($\varepsilon, \boldsymbol{\mu}, \boldsymbol{\alpha}$)	405.84 (±25.950)	427.07 (±36.057)	457.51 (±37.139)	2.05 (±0.601)	0.33 (±0.029)	1.05 (±0.077)
	($\varepsilon, \boldsymbol{\mu}, \boldsymbol{\alpha}, \mathbf{B}$)	342.72 (±19.319)	344.98 (±18.432)	416.58 (±34.368)	2.75 (±1.223)	0.41 (±0.107)	0.74 (±0.052)
Polyalkenes/acenes	property model	-	-	-	1.19 (±0.051)	0.72 (±0.006)	-
	(ε)	221.76 (±5.790)	226.32 (±4.108)	266.64 (±15.160)	5.58 (±0.215)	0.11 (±0.006)	1.89 (±0.115)
	($\varepsilon, \boldsymbol{\mu}$)	208.98 (±2.391)	223.67 (±5.989)	183.24 (±19.107)	1.42 (±0.037)	0.05 (±0.004)	0.99 (±0.119)
	($\varepsilon, \boldsymbol{\mu}, \boldsymbol{\alpha}$)	351.08 (±40.391)	362.12 (±45.521)	245.90 (±29.076)	1.80 (±0.055)	0.15 (±0.011)	0.67 (±0.105)
	($\varepsilon, \boldsymbol{\mu}, \boldsymbol{\alpha}, \mathbf{B}$)	315.56 (±30.366)	319.41 (±30.865)	322.64 (±69.106)	1.86 (±0.110)	0.15 (±0.012)	0.34 (±0.012)

Table S4: Mean absolute errors (MAEs) and standard deviations for various upscaled indirect models, that target properties from the def2-TZVP basis while predicting an effective Hamiltonian in the minimal basis, across different datasets (QM7, QM9, Polyenoic acid series and Polyalkenes/acenes series). The evaluated properties include MO energies (ε), MO energies upto the occupied orbital (ε_{occ}), HOMO-LUMO gap (Δ), dipole moment ($\boldsymbol{\mu}$), polarizability ($\boldsymbol{\alpha}$), and Mayer bond order ($\mathbf{B}$). The "property model" row represents the performance of property-specific models, while subsequent rows correspond to multi-task effective Hamiltonian models trained on increasing numbers of properties.

dataset	Model	ε (meV)	ε_{occ} (meV)	Δ (meV)	μ (mD/atom)	α (Å^3/atom)	$\mathbf{B}$ (-)
QM7	property model	-	-	-	1.17 (±0.083)	0.05 (±0.002)	-
	(ε)	190.28 (±3.572)	191.68 (±7.954)	334.18 (±18.097)	7.99 (±0.687)	0.46 (±0.008)	5.57 (±0.136)
	($\varepsilon, \boldsymbol{\mu}$)	208.17 (±17.067)	196.74 (±15.273)	350.71 (±34.760)	1.30 (±0.083)	0.58 (±0.007)	5.70 (±0.077)
	($\varepsilon, \boldsymbol{\mu}, \boldsymbol{\alpha}$)	287.18 (±6.735)	286.49 (±11.833)	422.72 (±23.997)	1.77 (±0.058)	0.11 (±0.011)	4.81 (±0.079)
	($\varepsilon, \boldsymbol{\mu}, \boldsymbol{\alpha}, \mathbf{B}$)	334.96 (±8.154)	325.46 (±12.115)	564.49 (±60.121)	1.74 (±0.131)	0.13 (±0.015)	3.85 (±0.029)
QM9	property model	-	-	-	2.60 (±0.037)	0.08 (±0.001)	-
	(ε)	291.54 (±5.282)	322.03 (±9.444)	618.50 (±38.350)	9.41 (±0.981)	0.44 (±0.006)	7.02 (±0.095)
	($\varepsilon, \boldsymbol{\mu}$)	302.36 (±14.796)	309.52 (±13.412)	347.39 (±14.861)	2.79 (±0.091)	0.59 (±0.005)	7.10 (±0.028)
	($\varepsilon, \boldsymbol{\mu}, \boldsymbol{\alpha}$)	380.74 (±2.502)	383.81 (±4.351)	564.55 (±22.754)	3.58 (±0.068)	0.15 (±0.001)	6.29 (±0.021)
	($\varepsilon, \boldsymbol{\mu}, \boldsymbol{\alpha}, \mathbf{B}$)	431.05 (±6.771)	427.80 (±5.037)	676.40 (±24.597)	3.43 (±0.060)	0.17 (±0.001)	5.26 (±0.023)
Polyenoics	property model	-	-	-	9.58 (±0.018)	1.40 (±0.002)	-
	(ε)	382.54 (±3.820)	378.34 (±14.926)	652.37 (±160.050)	10.90 (±2.764)	1.07 (±0.244)	10.66 (±0.218)
	($\varepsilon, \boldsymbol{\mu}$)	385.61 (±16.170)	322.26 (±14.732)	589.92 (±98.422)	2.52 (±0.454)	0.58 (±0.023)	10.36 (±0.022)
	($\varepsilon, \boldsymbol{\mu}, \boldsymbol{\alpha}$)	423.28 (±14.243)	386.49 (±10.025)	584.49 (±76.133)	4.11 (±0.846)	0.67 (±0.095)	9.13 (±0.040)
	($\varepsilon, \boldsymbol{\mu}, \boldsymbol{\alpha}, \mathbf{B}$)	476.94 (±7.786)	451.86 (±10.512)	507.08 (±32.446)	2.71 (±0.513)	0.44 (±0.040)	7.56 (±0.056)
Polyalkenes/acenes	property model	-	-	-	1.40 (±0.051)	0.87 (±0.006)	-
	(ε)	342.07 (±8.127)	340.42 (±19.074)	488.37 (±50.254)	4.62 (±0.451)	0.53 (±0.037)	6.63 (±0.101)
	($\varepsilon, \boldsymbol{\mu}$)	367.92 (±20.561)	323.30 (±23.621)	260.62 (±19.089)	1.41 (±0.160)	0.76 (±0.042)	7.19 (±0.049)
	($\varepsilon, \boldsymbol{\mu}, \boldsymbol{\alpha}$)	477.18 (±13.298)	439.32 (±13.204)	639.84 (±16.908)	1.79 (±0.222)	0.29 (±0.009)	5.85 (±0.049)
	($\varepsilon, \boldsymbol{\mu}, \boldsymbol{\alpha}, \mathbf{B}$)	468.72 (±13.682)	473.96 (±23.504)	512.71 (±13.478)	2.00 (±0.171)	0.30 (±0.010)	4.61 (±0.031)

References

(S1) Rupp, M.; Tkatchenko, A.; Müller, K.-R.; von Lilienfeld, O. A. Fast and Accurate Modeling of Molecular Atomization Energies with Machine Learning. *Phys. Rev. Lett.* **2012**, *108*, 058301.

(S2) Eldar, Y.; Lindenbaum, M.; Porat, M.; Zeevi, Y. Y. The farthest point strategy for progressive image sampling. *IEEE transactions on image processing* **1997**, *6*, 1305–1315.

(S3) Imbalzano, G.; Anelli, A.; Giofré, D.; Klees, S.; Behler, J.; Ceriotti, M. Automatic selection of atomic fingerprints and reference configurations for machine-learning potentials. *J. Chem. Phys.* **2018**, *148*, 241730.

(S4) Goscinski, A.; Principe, V. P.; Fraux, G.; Kliavinek, S.; Helfrecht, B. A.; Loche, P.; Ceriotti, M.; Cersonsky, R. K. scikit-matter : A Suite of Generalisable Machine Learning Methods Born out of Chemistry and Materials Science [version 2; peer review: 3 approved, 1 approved with reservations]. *Open Research Europe* **2023**, *3*.

(S5) Bartók, A. P.; Kondor, R.; Csányi, G. On representing chemical environments. *Phys. Rev. B* **2013**, *87*, 184115.

(S6) Veit, M.; Wilkins, D. M.; Yang, Y.; DiStasio, J., Robert A.; Ceriotti, M. Predicting molecular dipole moments by combining atomic partial charges and atomic dipoles. *The Journal of Chemical Physics* **2020**, *153*, 024113.

(S7) Ramakrishnan, R.; Dral, P. O.; Rupp, M.; von Lilienfeld, O. A. Quantum chemistry structures and properties of 134 kilo molecules. *Scientific Data* **2014**, *1*, 1–7.

(S8) Wilkins, D. M.; Grisafi, A.; Yang, Y.; Lao, K. U.; DiStasio, R. A.; Ceriotti, M. Accurate molecular polarizabilities with coupled cluster theory and machine learning. *Proc. Natl. Acad. Sci. USA* **2019**, *116*, 3401–3406.

(S9) Veit, M.; Wilkins, D. M.; Yang, Y.; DiStasio, R. A.; Ceriotti, M. Predicting Molecular Dipole Moments by Combining Atomic Partial Charges and Atomic Dipoles. *J. Chem. Phys.* **2020**, *153*, 024113.

(S10) Sun, Q.; Berkelbach, T. C.; Blunt, N. S.; Booth, G. H.; Guo, S.; Li, Z.; Liu, J.; McClain, J. D.; Sayfutyarova, E. R.; Sharma, S.; others PySCF: the Python-based simulations of chemistry framework. *Wiley Interdisciplinary Reviews: Computational Molecular Science* **2018**, *8*, e1340.

(S11) Stephens, P. J.; Devlin, F. J.; Chabalowski, C. F.; Frisch, M. J. Ab initio calculation of vibrational absorption and circular dichroism spectra using density functional force fields. *The Journal of physical chemistry* **1994**, *98*, 11623–11627.

(S12) Li, H.; Wang, Z.; Zou, N.; Ye, M.; Xu, R.; Gong, X.; Duan, W.; Xu, Y. Deep-learning density functional theory Hamiltonian for efficient ab initio electronic-structure calculation. *Nature Computational Science* **2022**, *2*, 367–377.

(S13) Kühne, T. D.; Iannuzzi, M.; Del Ben, M.; Rybkin, V. V.; Seewald, P.; Stein, F.; Laino, T.; Khaliullin, R. Z.; Schütt, O.; Schiffmann, F.; others CP2K: An electronic structure and molecular dynamics software package-Quickstep: Efficient and accurate electronic structure calculations. *The Journal of Chemical Physics* **2020**, *152*.

(S14) Perdew, J. P.; Burke, K.; Ernzerhof, M. Generalized Gradient Approximation made simple. *Phys. Rev. Lett.* **1996**, *77*, 3865.

(S15) Goedecker, S.; Teter, M.; Hutter, J. Separable dual-space Gaussian pseudopotentials. *Physical Review B* **1996**, *54*, 1703.

(S16) Featomic. 2023; `https://github.com/metatensor/featomic`.

(S17) Drautz, R. Atomic cluster expansion for accurate and transferable interatomic potentials. *Phys. Rev. B* **2019**, *99*, 014104.

(S18) Willatt, M. J.; Musil, F.; Ceriotti, M. Atom-density representations for machine learning. *J. Chem. Phys.* **2019**, *150*, 154110.

(S19) Nigam, J.; Willatt, M. J.; Ceriotti, M. Equivariant Representations for Molecular Hamiltonians and N -Center Atomic-Scale Properties. *J. Chem. Phys.* **2022**, *156*, 014115.

(S20) Paszke, A.; Gross, S.; Massa, F.; Lerer, A.; Bradbury, J.; Chanan, G.; Killeen, T.; Lin, Z.; Gimelshein, N.; Antiga, L.; others Pytorch: An imperative style, high-performance deep learning library. Advances in neural information processing systems. 2019; pp 8026–8037.
